# An Enhanced Plastic Optical Fiber-Based Surface Plasmon Resonance Sensor with a Double-Sided Polished Structure

**DOI:** 10.3390/s21041516

**Published:** 2021-02-22

**Authors:** Lian Liu, Shijie Deng, Jie Zheng, Libo Yuan, Hongchang Deng, Chuanxin Teng

**Affiliations:** 1 Guangxi Key Laboratory of Optoelectronic Information Processing, Guilin University of Electronic Technology, Guilin 541004, China; franco510@163.com (L.L.); shijie.deng@guet.edu.cn (S.D.); lbyuan@guet.edu.cn (L.Y.); hcdeng@guet.edu.cn (H.D.); 2 State Key Laboratory on Integrated Optoelectronics, College of Electronic Science and Engineering, Jilin University, Changchun 130012, China; zhengjie@jlu.edu.cn

**Keywords:** enhanced SPR sensor, plastic optical fiber, double side polished structure, refractive index measurement

## Abstract

An enhanced plastic optical fiber (POF)-based surface plasmon resonance (SPR) sensor is proposed by employing a double-sided polished structure. The sensor is fabricated by polishing two sides of the POF symmetrically along with the fiber axis, and a layer of Au film is deposited on each side of the polished region. The SPR can be excited on both polished surfaces with Au film coating, and the number of light reflections will be increased by using this structure. The simulation and experimental results show that the proposed sensor has an enhanced SPR effect. The visibility and full width at half maximum (FWHM) of spectrum can be improved for the high measured refractive index (RI). A sensitivity of 4284.8 nm/RIU is obtained for the double-sided POF-based SPR sensor when the measured liquid RI is 1.42. The proposed SPR sensor is easy fabrication and low cost, which can provide a larger measurement range and action area to the measured samples, and it has potential application prospects in the oil industry and biochemical sensing fields.

## 1. Introduction

Surface plasmon resonance (SPR) sensors are very sensitive to the small changes of refractive index (RI); after decades of development, they have been widely investigated in biochemical sensing fields, such as environmental monitoring, food safety, and disease diagnosis [1,2]. In recent years, optical fiber-based SPR sensors have attracted more and more attention because of the advantages of low cost, compact size, and being available for real time and in situ monitoring [3,4,5]. In 1993, C. Jorgenson et al. [6] reported the optical fiber-based SPR sensor for the first time, since then, this kind of SPR sensor has obtained numerous researches. Different from the prism-based SPR sensors, the optical fiber-based SPR sensors often work in the wavelength modulation mode, and the fiber core or cladding replaces the glass prism to couple the incident light to the metal film. In order to excite the surface plasma wave, the p-polarized light energy has to be entered into the metal film in the form of an evanescent wave. To date, many types of fiber-based SPR sensors have been proposed, which are designed to generate the evanescent wave, for example, the tapering fiber-based SPR sensors [7], the side-polishing fiber-based SPR sensors [8], the tip-polishing fiber-based SPR sensors [9], the hetero-core fiber-based SPR sensors [10], the tilted fiber Bragg grating-based SPR sensors [11], the long-period fiber grating-based SPR sensors [12], and so on. Different kinds of optical fibers were also employed to design the SPR sensors, including the single-mode fibers, the multimode fibers, the microstructure fibers, and so on. The single-mode fibers will become very brittle after tapering or side-polishing process due to their small fiber core diameter. Although the tilted fiber Bragg grating and the long-period fiber grating could excite the SPR effect without destroying the fiber structure, their sensitivity is relatively low and the fabrication process is complicated. Compared with the single-mode fiber, the multimode fiber has a large core diameter, which could maintain a good mechanical strength even after the side-polishing or tapering process; in addition, the coupling efficiency between the multimode fiber and the broadband light sources is higher. Although the width of the SPR spectrum for the multi-mode fiber is broader because of the large number of guided modes, after the fiber structure or the multilayer material optimization, the spectral width can be narrowed [13,14].

Plastic optical fiber (POF) is a kind of optical fiber made from light-transmitting polymeric materials. Compared with the glass counterparts, the POFs have the features of large diameter, low cost, easy operation, good flexibility, and low attenuation in the visible wavelength. In recent years, the POFs-based SPR sensors have attracted more and more attention. Many POFs-based SPR sensors have been reported [15,16,17,18,19,20,21], for example, Jing et al. developed a side-polished macrobending POF-based SPR for RI sensing [22]. A. Ariadny et al. presented a U-shape POF-based SPR sensor for E. coli bacteria detection [23]. S. Cao et al. proposed a highly sensitive SPR biosensor based on a low-index POF [24]. N. Cennamo et al. investigated the effects of different photoresist buffer layers on the POF-based SPR sensor performance [25], and a POF-SPR-based magnetic field sensor [26] and a fluorescent POF-based SPR sensor [27] were also proposed by the same group. In addition, a gold-supported graphene composite film-coated POF-based SPR sensor for dopamine detection was also reported [28]. The recent developments of the POFs-based SPR sensors have demonstrated that they are playing a more and more important role in the SPR sensing fields. However, due to the multimode characteristics of POF, the resonance spectrum of the POF-based SPR sensors is broader, especially for the high surrounding RI, which is adverse to the high precision measurement. Therefore, some researchers try to optimize the POFs-based SPR sensor performance, for example, N. Cennamo et al. introduced a tapered structure to the side polished POF-based SPR sensor as the modal filter to optimize the SPR spectrum [29], and they also compared the sensing performances for the D-shaped tapered POFs-based SPR sensors with different taper ratios [30]. Teng et al. [31] optimized the sensing performance of the side-polished U-shape POF-based SPR sensor. However, cascading the tapered structure will increase the sensor size and reduce the mechanical strength of the device; the usage of a U-shape structure will introduce more bending loss to the sensor. Furthermore, the visibilities of SPR spectra for these structures are also reduced severely at high measured RI.

In this paper, we propose an enhanced POF-based SPR sensor using a double-sided polished structure. The structure is fabricated by polishing two sides of the POF symmetrically along with the fiber axial; after coating the Au film on both sides of the polished region, the SPR probe can be obtained. The number of light reflections will be increased by using this structure. The simulation and experimental analyses are carried out, and the RI sensing performances for the single-sided and double-sided polished probes are compared experimentally. Experimental results show that the proposed sensor has an obvious SPR effect even for the high environment RI. A sensitivity of 4284.8 nm/RIU is obtained for the proposed sensor when the liquid RI is 1.42. The proposed sensor probe is easy fabrication, low cost, and possesses a good mechanical strength, which can provide a larger measurement range and action area to the measured samples without increasing the sensor length.

## 2. Sensor Structure and Operation Principle

**Figure 1:**
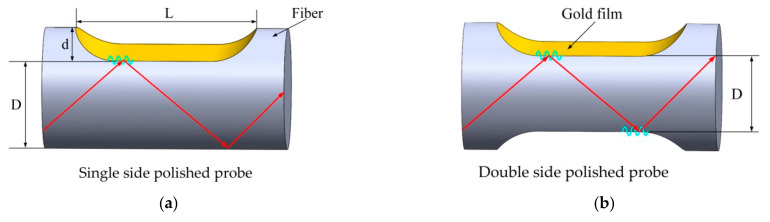
The schematics of the (**a**) single and (**b**) double-sided polished fiber surface plasmon resonance (SPR) probes. *D* is the remaining diameter of the side polished region, *L* is polished region length, and *d* is the polished depth.

The single and double-sided polished structures of the POF-based SPR sensor probes are shown in Figure 1.

For the single-sided polished probe, the SPR is excited just at one side of the polished region, while for the double-sided polished fiber probe, whose polished regions are symmetrically located on both sides of the POF along with the fiber central axis, the SPR can be excited at both sides of the polished regions. For the light (meridional ray) propagating through the double-sided polished SPR sensing region with an angle *θ* from the normal to core–metal layer interface, the total number of light reflections *N(θ)* can be expressed as:

(1) $$N\left(\theta \right)=\frac{L}{D\tan\theta }$$

where *L* is the SPR sensing region length, *D* is the remaining diameter of the side polished region, and $N\left(\theta \right)$ equals to the integral part of its value. Obviously, the total number of light reflections for the double-sided polished SPR probe is nearly twice that of the single-sided polished one.

The Krestchmann configuration was employed to analyze the proposed POF-based SPR sensor. As shown in Figure 1, a three-layer model was used to simulate the SPR sensor working in the wavelength interrogation mode. The first layer is the POF core made of polymethyl methacrylate (PMMA) with RI of 1.49; the second layer is the metal layer with dielectric function *ε_m_* in the Lorentz–Drude model [32] and thickness of *d_2_*; and the third layer is the measured medium with the RI of *n_ex_*. The normalized transmitted power (for meridional rays) of p-polarized light $\left(P_{trans}\right)$ for the double-sided polished POF-based SPR sensor can be expressed as [33]:

(2) $$P_{trans}=\frac{\int_{\theta_{c}}^{\pi /2}R^{N\left(\theta \right)}P\left(\theta \right)d\theta }{\int_{\theta_{c}}^{\pi /2}P\left(\theta \right)d\theta }$$

where $\theta_{c}=\sin^{-1}\left(n_{co}/n_{cl}\right)$ is the critical angle of total reflection, and $n_{co}$ and $n_{cl}$ are the RIs of POF core and cladding ($n_{cl}=1.41$), respectively. $P\left(\theta \right)$ is the modal power corresponding to the incident angle θ, which can be expressed as:

(3) $$P\left(\theta \right)=\frac{n_{c}^{2}\sin\theta \cos\theta }{{\left(1-n_{c}^{2}\cos^{2}\theta \right)}^{2}}$$

**Figure 2:**
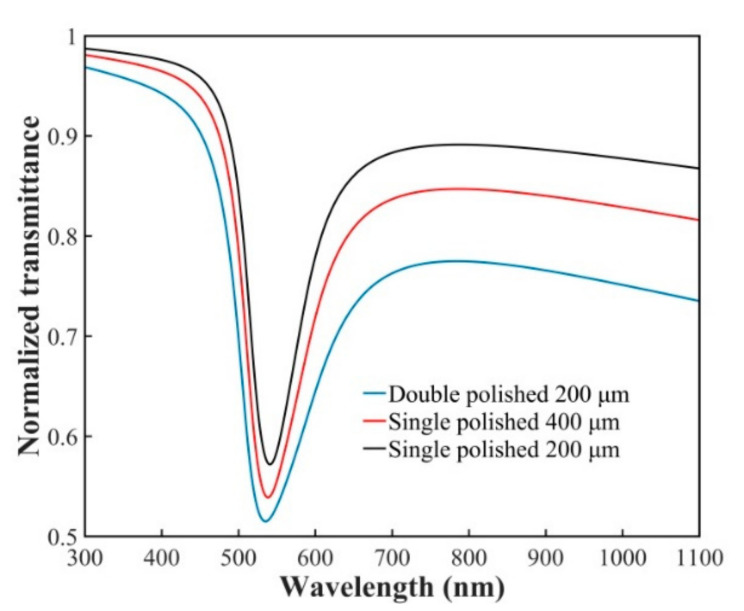
The simulation results of the normalized transmitted spectra for the single and double-sided polished fiber SPR probes with different polished depths in the external medium with refractive index (RI) of 1.34.

Figure 2 shows the normalized transmitted spectra for the single-sided polished fiber SPR probes with polished depths of 200 and 400 μm, and the double-sided polished one with a polished depth of 200 μm. The polished length *L* is 5 mm, the thickness of the Au film *d*_2_ = 50 nm, and the RI for the measured medium is 1.34. It can be seen that the SPR peak for the double-sided polished fiber probe is the deepest, which shows an enhanced SPR effect.

**Figure 3:**
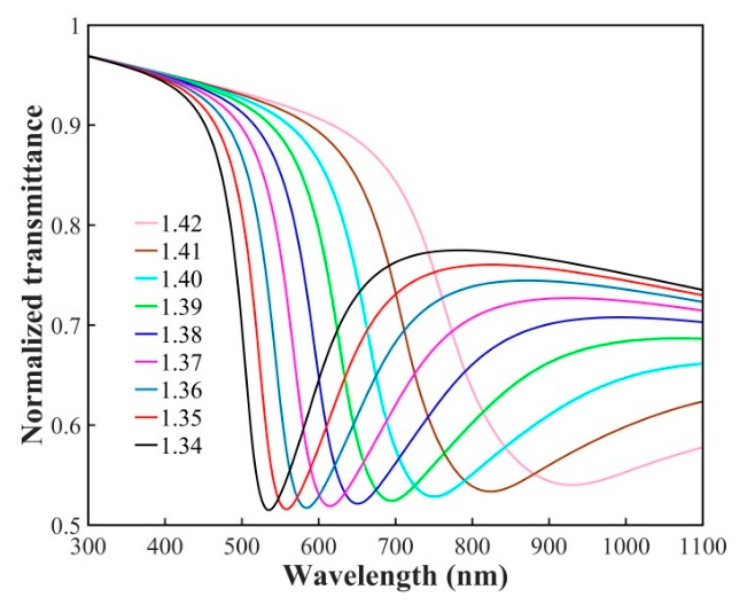
Simulation results of the double-sided polished SPR probe with an RI sensing range of 1.34–1.42.

Figure 3 shows the simulation results of the double-sided polished SPR probe for RI sensing with an RI range of 1.34–1.42. According to Figure 3, it can be seen that as the RI increases, the SPR peak has a red shift, the width of the SPR peak becomes broader, and the transmitted intensity of the sharpest SPR peak decreases.

## 3. Sensor Probe Fabrication

**Figure 4:**
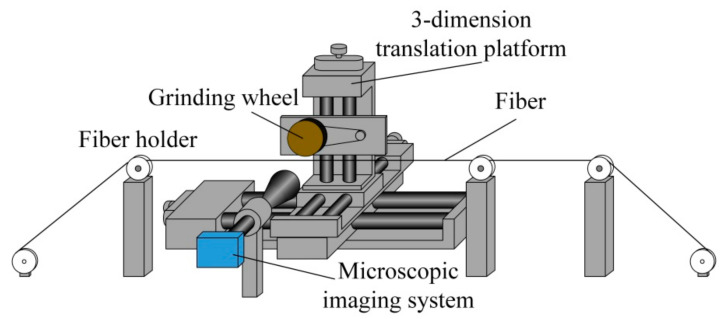
The schematic diagram of the wheel polishing system.

The proposed double-sided polished sensor probe was made of commercially available step-index POF (Eska, SK40) with a core material of PMMA, diameter of 980 μm, and RI of 1.49. The cladding material of the fiber is fluorinated polymer with thickness of 10 μm and RI of 1.41. A wheel polishing system was used to fabricate the double-sided polished POF. As shown in Figure 4, the system consists of a three-dimensional displacement platform, two fiber holders, a grinding wheel, a camera, and a computer.

**Figure 5:**
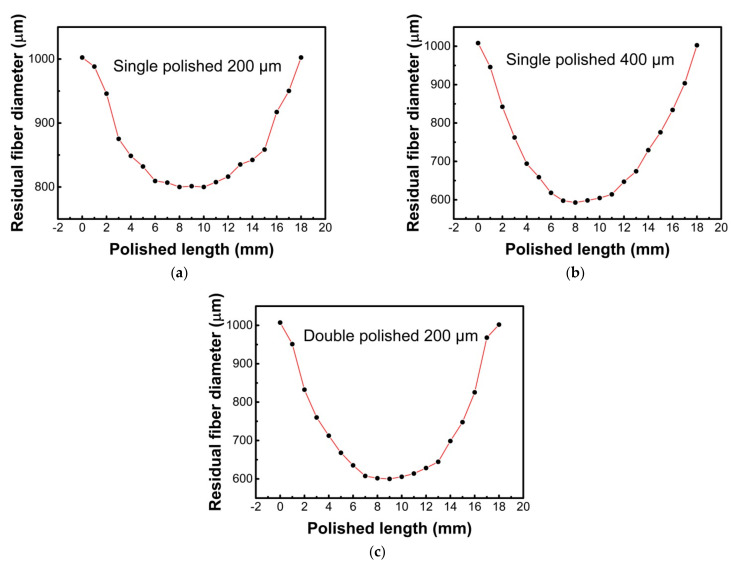
The structural parameters for the single and double-sided polished plastic optical fiber (POF) probes. For the single-sided polished SPR probe with polished depths of 200 μm (**a**) and 400 μm (**b**), and the double-sided polished SPR probe with a polished depth of 200 μm (**c**).

During the polishing process, the POF is fixed by the fiber holder and kept in a tensional state. The rotatable grinding wheel pasted with an abrasive paper (7000-mesh) is used to grind the POF. The grinding wheel is connected to the three-dimensional micro-displacement stage, which could be controlled by a computer. The rotating speed of the grinding wheel is also controlled by the computer. A microscopic imaging system is used to monitor the structural parameters of the fiber during the fabrication process. The polished depth and length of the fiber could be controlled by adjusting the three-dimensional micro-displacement stage and the rotating speed of the grinding wheel, respectively. In order to get the double-sided polished structure, after polishing one side, the fiber needs to be rotated 180° along with the fiber axis, and then the same grinding process is implemented to the other side of the fiber. Figure 5 shows the structural parameters for the single and double-sided polished POF probes fabricated in this study.

**Figure 6:**
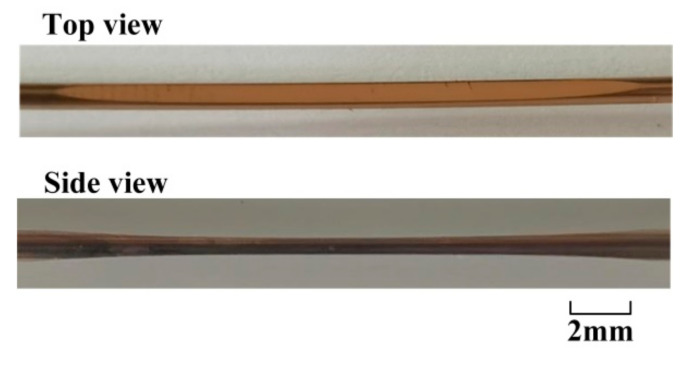
The photos of the double-sided polished fiber SPR probe.

In order to get a smooth polishing surface, after the grinding process, the aluminium oxide polishing paste was used to polish the grinding region. In the end, a layer of Au film was deposited on the polished region by the plasmon sputtering technique; by adjusting the sputtering current and time, the Au layer thickness can be changed. In this process, a film thickness detector was used to monitor the Au film thickness. After coating one side of the polished region, the POF needs to be rotated 180° to coat the other side. In our experiment, the deposition thickness of the Au layer is 50 nm with an accuracy of ±3 nm. The photo of the double-sided polished SPR probe is shown in Figure 6.

## 4. Experimental Results and Discussions

**Figure 7:**
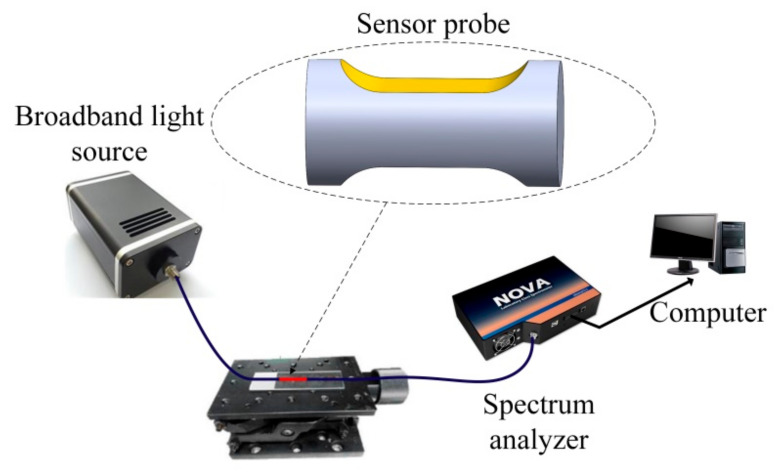
The schematic illustration of the experiment setup.

The schematic illustration of the experiment setup is shown in Figure 7. A broadband light source (ideaoptics HL2000, Shanghai, China, wavelength range of 360–2500 nm) was employed to provide a broadband transmission light, and the transmitted light spectrum was detected by a spectrum analyzer (ideaoptics NOVA, Shanghai, China, wavelength range of 325–1100 nm and resolution of 0.77 nm). The glycerol–water solutions with different volume fractions were prepared as the measured samples. The RIs for the glycerol–water solutions ranged from 1.34 to 1.42 with an RI step of 0.01. An Abbe refractometer was used to measure the refractive indices of the solutions. The experiment was carried out at a room temperature of 20 ℃.

**Figure 8:**
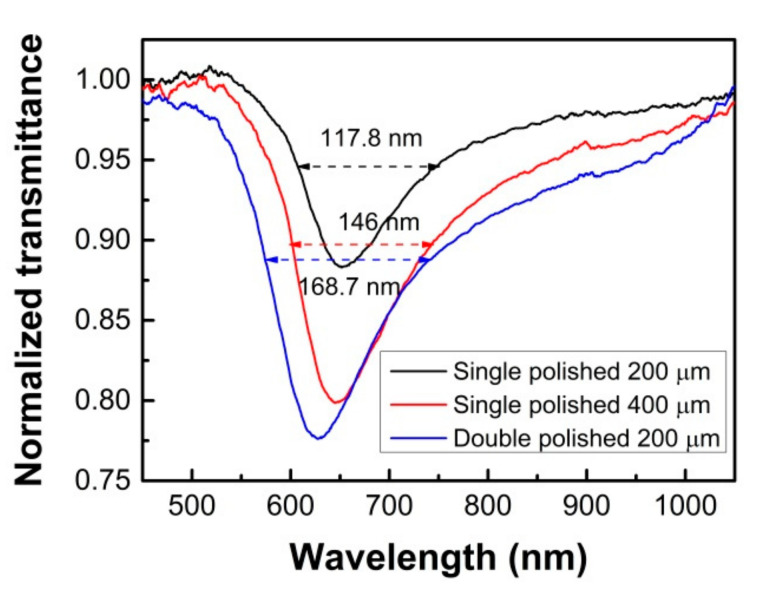
The normalized transmission spectra for the single and double-sided polished POF-based SPR probes in the external medium with RI of 1.34.

Figure 8 shows the normalized transmission spectra for the proposed single and double-sided polished POF SPR probes in the measured analyte with RI of 1.34. The SPR transmission spectra were normalized to the air spectrum. It is found that as the single side polished depth increases from 200 μm to 400 μm, the transmitted intensity of sharpest SPR peak on the spectrum changes significantly from 88.4% to 79.9%. The same system and process repeats for the double-sided polished SPR probe; the transmitted intensity of the sharpest SPR peak changes to 77.6%, which means a stronger SPR excitation effect. The experimental results have a similar trend to the simulation results, but the wavelength location and the transmittance value of the SPR peak are somewhat different. This may be because there were thousands of propagation modes in the POF-based SPR probes, in our simulation, however, only the meridional rays were taken into account, while the skew rays, which could also excite the SPR and broaden the resonance peak, were not taken into account. From Figure 8, it can be also obtained that the full width at half maximum (FWHM) is a little broadened for the double-sided polished probe in the measured analyte with an RI of 1.34. For the single-sided polished probes with polished depths of 200 and 400 μm, the FWHM are 117.8 and 146 nm, respectively, and 168.7 nm for the double-sided polished probe. The FWHM is measured to 100% normalized transmission level, which could determine the accuracy of the detection, and a better accuracy could be achieved from a narrower FWHM.

**Figure 9:**
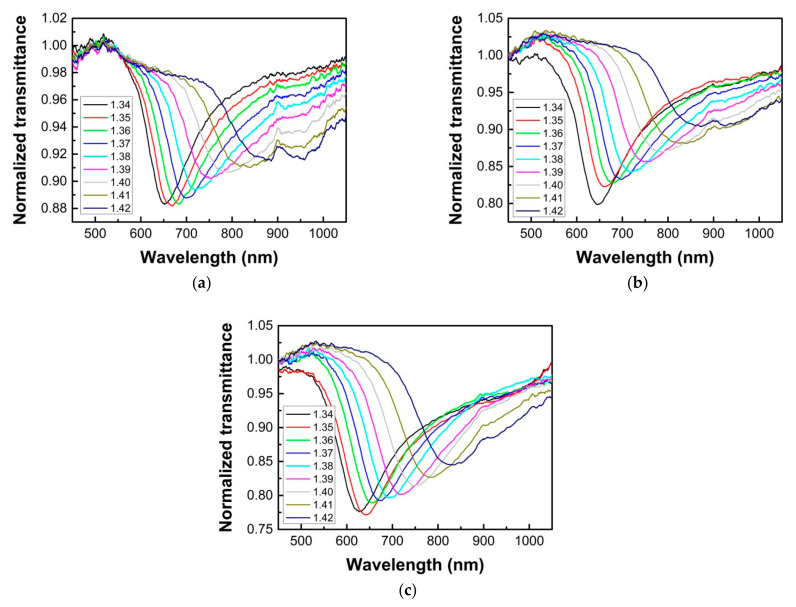
The SPR transmission spectra in glycerol–water solutions with different RIs for the single-sided polished SPR probe with polished depths of 200 μm (**a**) and 400 μm (**b**), and the double-sided polished SPR probe with a polished depth of 200 μm (**c**).

The SPR sensing performances for the fabricated single and double-sided polished POF probes are shown in Figure 9. It is found from Figure 9a–c that the SPR peak for each probe has a red shift as the RI increases, and the FWHM will increase as the RI increases, which agrees with the simulation results. It can also be seen that for the single-sided polished SPR probe with polished depths of 200 μm, the broadening of the resonance spectrum and the reduction in resonance effect are very severe, and it has been difficult to determine an accurate location of the SPR peak when the measured liquid RI is 1.42. For the single-sided polished SPR probe with a polished depth of 400 μm, the situation has some improvement, but the resonance effect is also declined obviously. However, for the double-sided polished SPR probe, the visibility of the spectrum is improved obviously, the broadening of the spectrum is not very severe, and the resonance effect is not greatly declined even for the higher measured liquid RI, therefore, it can still be easy to determine the SPR peak location more precisely.

**Figure 10:**
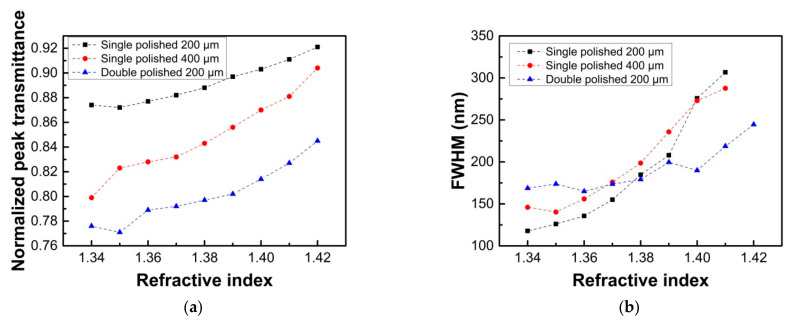
The transmitted intensity of the sharpest SPR peak (**a**), and the full width at half maximum (FWHM) of normalized transmission spectra (**b**).

Figure 10 shows the transmitted intensity of the sharpest SPR peak and the FWHM of the normalized transmission spectra for the single and double-sided polished probes in the external medium with RI of 1.34–1.42. Results show that the double-sided polished probe has the minimum transmitted intensity for the sharpest SPR peak, and there is still 84.5% transmitted intensity when the measured liquid RI is 1.42. Results also show that the changes of FWHM for the double-sided polished probe are not very obvious for the measured liquid in the RI range of 1.34–1.38, which is different from the situations of the single polished probes. In addition, in the RI range of 1.38–1.42, the FWHM for the double-sided polished probe becomes the narrowest, while for the single polished ones, it has been difficult to determine the FWHM accurately when the RI is 1.42. Therefore, the results indicate the proposed sensor has an enhanced SPR effect.

**Figure 11:**
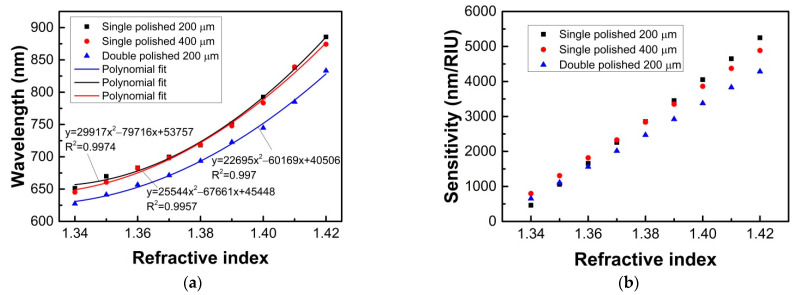
Resonance wavelength peaks as a function of the RI ranging from 1.34 to 1.42 (**a**), and the sensitivities (**b**) for the single and double-sided polished sensor probes.

The sensitivities for these probes were also evaluated, which can be defined as:

(4) $$S=\delta \lambda /\delta n_{ex}\;$$

where *S* is the sensitivity, δλ is the changes of resonance wavelength, and $\delta n_{ex}$ is the RI changes of the measured liquid. Figure 11 shows the wavelength shifts of the resonance peaks and the sensitivities for the probes in the RI range of 1.34–1.42. It can be found that the shifts of resonance peaks to the external RI are nonlinear, and the sensitivities increase with the RI varying from 1.34 to 1.42. It is also found that there are some decreases in sensitivity for the double-sided polished SPR probe for higher measured liquid RIs. When the liquid RI is 1.42, the sensitivity for the double-sided polished SPR probe can reach 4284.8 nm/RIU. Although the sensitivity for the double-sided polished SPR probe is a little lower than those of the single polished ones, it has been enough for the liquid RI detection. In addition, by considering its merit of enhanced SPR effect in the higher measured RI, the proposed sensor may have potential applications in the oil industry where the measured samples have high RIs.

**Figure 12:**
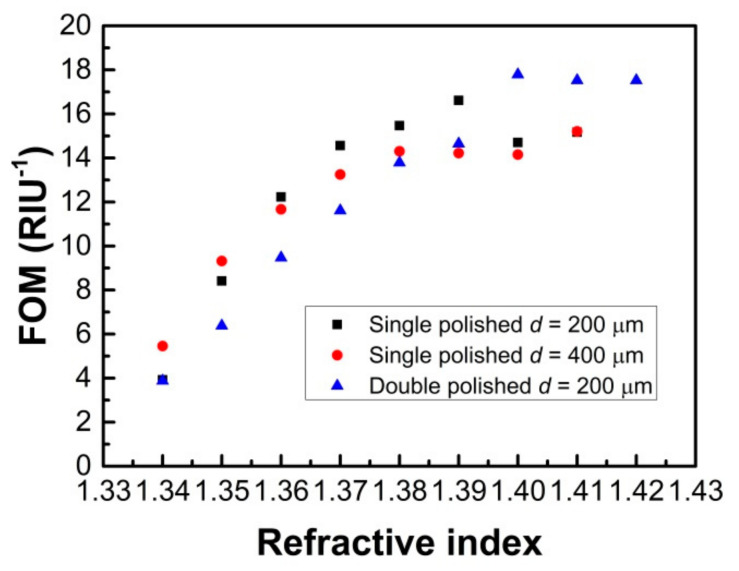
The figure of merit (FOM) for the single and double-sided polished sensor probes.

The figures of merit (FOMs) for the single and double-sided polished SPR probes are also analyzed, which are determined by the ratio of sensor sensitivity to FWHM [3]. As shown in Figure 12, it can be seen that the double-sided polished probe has a higher FOM for the high measured RI.

The sensing performance comparison for the proposed sensor probe and other POFs-based SPR sensor probes with different structures is shown in Table 1. It can be seen that the proposed sensor can provide a comparable sensitivity and a larger measurement range. The FWHM at lower measured RI for the proposed sensor probe is also comparable, but the FWHM for the measured RI higher than 1.41 were not provided by other references.

## 5. Conclusions

We proposed an enhanced POF-based SPR sensor by employing a double-sided polished structure. The structure was fabricated by polishing both sides of the POF symmetrically along with the fiber axis. This structure can excite the SPR effect on both sides of the fiber–Au film interfaces, which can increase the number of light reflections and bring an enhanced effect. The RI sensing performance comparison between the proposed sensor probe and the single-sided polished ones were implemented; both the simulation and experimental results showed that the double-sided polished probe had an enhanced SPR effect in the external medium with RI of 1.34. The SPR peak depth, FWHM, sensitivity, and FOM for the sensor probes were evaluated experimentally. Results showed that the double-sided polished probe had the deepest SPR peak and its FWHMs and FOMs for the high sample RIs were also the best. Especially, the proposed probe can provide a better visibility even for the high measured RI, which can provide a more accurate measurement. A sensitivity of 4284.8 nm/RIU was obtained for the probe when the liquid RI was 1.42. Besides the enhanced effect, the proposed sensor is easy fabrication, low cost, and possesses a good mechanical strength, which has potential applications in the oil industry, where the large measurement range or high sample RI detection is needed.

## Figures and Tables

**Table 1:** Performance comparison for different types of plastic optical fibers (POFs)-based surface plasmon resonance (SPR) sensors.

Sensor Structure	S (nm/RIU)	Full Width at Half Maximum (nm)	Dynamic Range	Ref.
D-shaped POF with buffer layer	2500	~150	1.332–1.418	[18]
D-shaped POF	1325	~200	1.332–1.372	[19]
Etched POF	1600	~154	1.3353–1.3653	[20]
U-Bent POF	1040	~100	1.33–1.361	[21]
Side polished low-index POF	22,779	~250	1.3–1.335	[24]
Side polished tapered POF	1700	~90	1.332–1.381	[30]
Side polished macrobending POF	4503.8	~138.6	1.335–1.41	[31]
Double side polished POF	4284.8	~168.7	1.34–1.42	Our work

## Data Availability

The data presented in this study are available on request from the corresponding author.
